# Disrupted auto-regulation of the spliceosomal gene *SNRPB* causes cerebro–costo–mandibular syndrome

**DOI:** 10.1038/ncomms5483

**Published:** 2014-07-22

**Authors:** Danielle C. Lynch, Timothée Revil, Jeremy Schwartzentruber, Elizabeth J. Bhoj, A. Micheil Innes, Ryan E. Lamont, Edmond G. Lemire, Bernard N. Chodirker, Juliet P. Taylor, Elaine H. Zackai, D. Ross McLeod, Edwin P. Kirk, Julie Hoover-Fong, Leah Fleming, Ravi Savarirayan, Care4Rare Canada, Kym Boycott, Alex MacKenzie, Michael Brudno, Dennis Bulman, David Dyment, Jacek Majewski, Loydie A. Jerome-Majewska, Jillian S. Parboosingh, Francois P. Bernier

**Affiliations:** 1 Department of Medical Genetics, University of Calgary, Calgary, Alberta, Canada T2N 4N1; 2 Department of Human Genetics, McGill University, Montréal, Quebec, Canada H3A 1B1; 3 McGill University and Génome Québec Innovation Centre, Montréal, Quebec, Canada H3A 0G1; 4 Division of Genetics, The Children’s Hospital of Philadelphia, Philadelphia, Pennsylvania 19104, USA; 5 Alberta Children’s Hospital Research Institute for Child and Maternal Health, Calgary, Alberta, Canada T3B 6A8; 6 Division of Medical Genetics, Department of Pediatrics, University of Saskatchewan, Saskatoon, Saskatchewan, Canada S7N 0W8; 7 Department of Pediatrics and Child Health, University of Manitoba, Winnipeg, Manitoba, Canada R3A 1S1; 8 Department of Biochemistry and Medical Genetics, Faculty of Medicine, University of Manitoba, Winnipeg, Manitoba, Canada R3A 1S1; 9 Genetic Health Service, Auckland 1142, New Zealand; 10 Sydney Children’s Hospital, Randwick, New South Wales 2031, Australia; 11 School of Women’s and Children’s Health, University of New South Wales, Randwick, New South Wales 2031, Australia; 12 Greenberg Center for Skeletal Dysplasias, McKusick-Nathans Institute of Genetic Medicine, Johns Hopkins University, Baltimore, Maryland 21287, USA; 13 National Human Genome Research Institute, National Institutes of Health, Bethesda, Maryland 20892, USA; 14 Department of Pediatrics, McGill University Health Centre, Montreal, Quebec H3Z 2Z3, Canada; 15 Department of Pediatrics, McGill University, Montreal Children’s Hospital, Montreal, Quebec, Canada H3H 1P3; 16 Children’s Hospital of Eastern Ontario Research Institute, University of Ottawa, 401 Smyth Road, Ottawa, Ontario, Canada K1H 8L1.; 17 Department of Computer Science, University of Toronto, Ontario, Canada.; 18 Donnelly Centre and Banting and Best Department of Medical Research, University of Toronto, Toronto, Ontario, Canada.; 19 Steering committee members and their affiliations appear at the end of the paper; 20 These authors contributed equally to this work

## Abstract

Elucidating the function of highly conserved regulatory sequences is a significant challenge in genomics today. Certain intragenic highly conserved elements have been associated with regulating levels of core components of the spliceosome and alternative splicing of downstream genes. Here we identify mutations in one such element, a regulatory alternative exon of *SNRPB* as the cause of cerebro–costo–mandibular syndrome. This exon contains a premature termination codon that triggers nonsense-mediated mRNA decay when included in the transcript. These mutations cause increased inclusion of the alternative exon and decreased overall expression of *SNRPB*. We provide evidence for the functional importance of this conserved intragenic element in the regulation of alternative splicing and development, and suggest that the evolution of such a regulatory mechanism has contributed to the complexity of mammalian development.

## Abstract (web summary)

Cerebro–costo–mandibular syndrome, CCMS, is a severe human multiple malformation disorder. Here, the authors report that mutations in *SNRPB* disrupt the normal regulation of alternative splicing at this gene, and in so doing, may be responsible for the development of CCMS.

## Graphical abstract

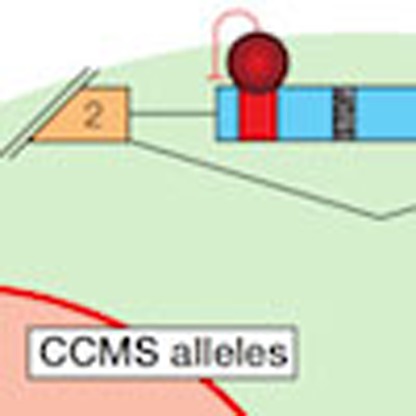

Although only 1.5% of the human genome consists of regions that are translated into proteins, a higher proportion (5–7%) has been shown to be under evolutionary constraint^1^,^2^. These non-coding conserved elements (NCEs) have been subclassified by somewhat arbitrary length and conservation criteria, and include ultraconserved^3^ and highly conserved^4^ elements. The finding that many NCEs exhibit a higher level of conservation^5^ and constraint^6^ than protein-coding sequences initially perplexed the genomics community. The elucidation that some NCEs have functional roles as long range enhancers of flanking genes, splicing regulators, functional co-activators^7^,^8^,^9^ and their frequent association with developmental genes with the potential to regulate spatiotemporal expression^7^,^10^, imply a largely regulatory role. The evolution of these complex regulatory networks may therefore have underpinned the emergence of our organismal complexity^6^,^11^.

Evidence continues to emerge of a critical relationship between NCEs and alternative splicing (AS), the mechanism by which over 95% of human multi-exon genes create additional protein diversity^12^,^13^. Intragenic NCEs are preferentially associated with genes involved in pre-mRNA splicing^3^, and are also often involved in the regulation of the expression of this class of genes by coupling AS with nonsense-mediated decay (NMD)^14^,^15^,^16^. Many genes involved in pre-mRNA splicing have ultra and highly conserved NCEs containing premature termination codons (PTCs), which can be alternatively spliced into the mature mRNAs to induce NMD in order to auto-regulate their expression^14^,^15^,^16^. These AS-NMD-mediated mechanisms are presumed to be crucial to the homeostatic maintenance of the core spliceosome components and the regulation of AS in a spatiotemporally specific manner by gene auto- and cross-regulation^17^,^18^.

Here we present mutations in a highly conserved, alternative PTC-containing exon of the small nuclear ribonucleoprotein polypeptides B and B1 (*SNRPB*) gene (Fig. 1) as the cause of cerebro–costo–mandibular syndrome (CCMS), a human multiple malformation disorder characterized by posterior rib gaps and Pierre Robin sequence (micrognathia, glossoptosis and cleft palate). This finding provides biological evidence of a direct link between conserved genomic elements, regulation of AS and human development, and therefore novel insight in the regulatory and developmental role of NCEs.

## Results

**Figure 1:**
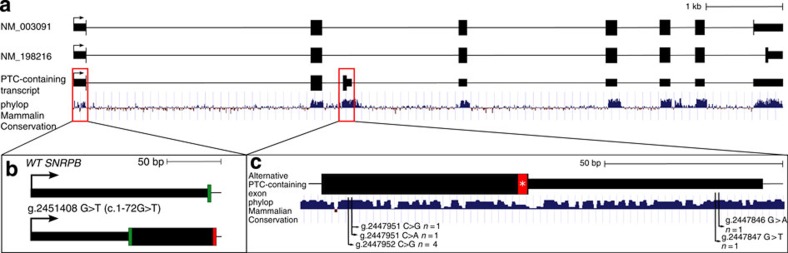
*SNRPB* mutations in CCMS. (**a**) The transcript isoforms encoding SmB (NM_003091), SmB′ (NM_198216), and the alternative PTC-containing transcript. (**b**) One patient (D II-1) had a mutation in the 5′ UTR predicted to introduce an upstream out-of-frame TIS, leading to a PTC after 25 amino acids (SOM text). The green boxes represent translation initiation codons, and the red box and asterisk represents a translation termination codon. (**c**) Five mutations within the alternative PTC-containing exon were identified in CCMS patients. These cluster at four nucleotides at the 5′ and 3′ ends of the exon within blocks of high conservation. WT, wild type.

A combination of whole-exome sequencing and Sanger sequencing was used to identify causative mutations in a cohort of 10 unrelated families with CCMS, a rare genetic disorder characterized by micrognathia and posterior rib gaps^19^ (Supplementary Fig. 1). All patients have typical features of CCMS except one patient (Family D) who had a more severe disease (Supplementary Methods, Supplementary Fig. 2 and Supplementary Table 1). Nine of the 10 patients had heterozygous regulatory mutations in *SNRPB*. Overall, six distinct, novel mutations in *SNRPB* were identified. Five mutations are within the alternative PTC-containing exon (chr20:g.2447838_2447961) of *SNRPB*. These mutations cluster at the 5′ and 3′ ends of this exon within areas of high conservation (Fig. 1c). A single patient had a 5′ untranslated region (UTR) mutation, which is predicted to introduce an out-of-frame translation initiation site (TIS) leading to a stop codon after 25 amino acids (Fig. 1b). In the *SNRPB-*positive families, mutation analysis confirms that CCMS is an autosomal dominant disorder. We observed a high rate of *de novo* mutations and two instances of non-penetrance. One individual with classic CCMS was negative for sequence or copy-number variants in the coding and UTRs of *SNRPB*.

**Figure 2:**
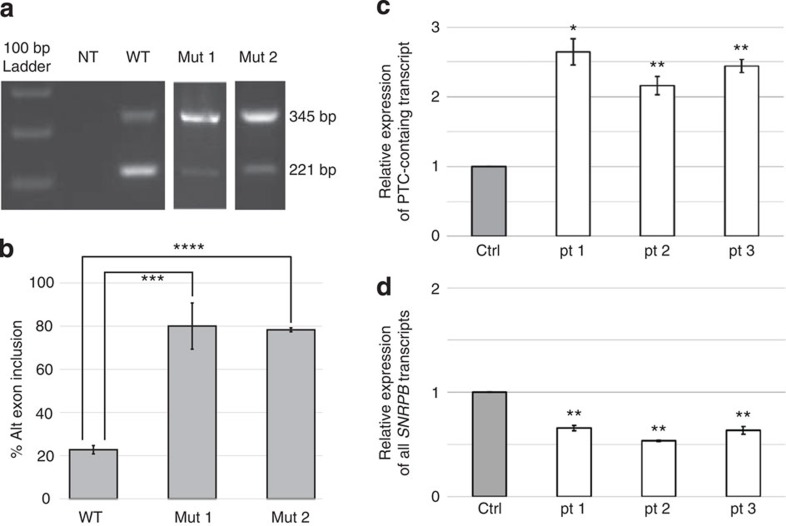
*SNRPB* mutations in the alternative (alt) PTC-containing exon cause increased exon inclusion. (**a**,**b**) Cloning of the alternative exon with either the chr20:g.2447951C>G (mut1) or chr20:g.2447847G>T (mut2) mutation into a splicing minigene reporter and transfection into HEK297 cells shows 78% and 80% exon inclusion, respectively, compared with 23% for the wild-type (WT) sequence. Results shown are from one representative experimental replicate of three. A 100-bp DNA ladder was used as a size marker in **a**. NT, no template control. (**c**) Patient fibroblasts with the chr20:g.2447951C>G (pt 1), chr20:g.2447952C>G (pt 2) and chr20:g.2447847G>T (pt 3) mutations show increased expression of the PTC-containing transcript by qRT–PCR. (**d**) The same three patients show decreased total *SNRPB* expression by qRT–PCR. In **c**,**d**, the grey columns represent the normalized average expression from three anonymous controls (Ctrl). The experiment was performed three times. For **b**–**d**, statistical significance was determined with a Student’s *t*-test. Bars indicate s.d. * indicates 0.005<*P*<0.05, ** indicates 0.0005<*P*<0.005 with *** indicating *P*<0.001 and ****P<0.0001.

*SNRPB* encodes the protein isoforms SmB and SmB′, which are core components of the U1, U2, U4/U6 and U5 small ribonuclear protein (snRNP)^20^ subunits of the major spliceosome. The highly conserved alternative exon within the second of six introns in *SNRPB* contains a PTC and has been shown to auto-regulate *SNRPB* levels through NMD^18^. The alternate exon, which has a sub-optimal 5′ splice site, is less frequently included when U1 snRNP levels are low as a result of SmB/B′ depletion^18^. Conversely, it is more frequently included with SmB/B′ overexpression^16^. We hypothesized that the mutations identified within this exon would alter the homeostatic balance between the coding full-length mRNA and alternative exon-containing transcripts targeted for degradation. Thus, we determined the effect of two of the alternative exon mutations using a splicing reporter minigene assay^21^. In the presence of the wild-type exon, 23% of all transcripts include this alternative exon, while introduction of either the chr20:g.2447951C>G or chr20:g.2447847G>T mutation shifts the proportion to 78% and 80%, respectively (Fig. 2a,b). Inclusion of the alternative PTC-containing exon was also assessed by quantitative reverse transcription PCR (qRT–PCR) in patient fibroblasts with the chr20:g.2447951C>G, chr20:g.2449752C>G, and chr20:g.2447847G>T mutations. Expression of the PTC-containing transcript increased, whereas overall expression of *SNRPB* decreased compared with control cells (Fig. 2c,d).

## Discussion

**Figure 3:**
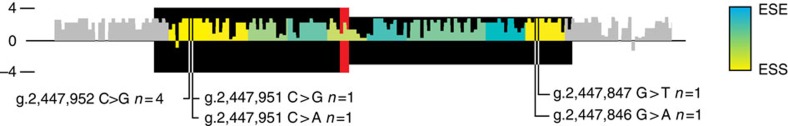
CCMS mutations overlap with highly conserved ESSs. The bar height shows mammalian PhyloP conservation scores within and flanking the alternative PTC-containing exon. The colours represent the strength of exonic splicing enhancers (ESEs) and silencers (ESSs) identified by deletion mutagenesis of miniSmB (17).

**Figure 4:**
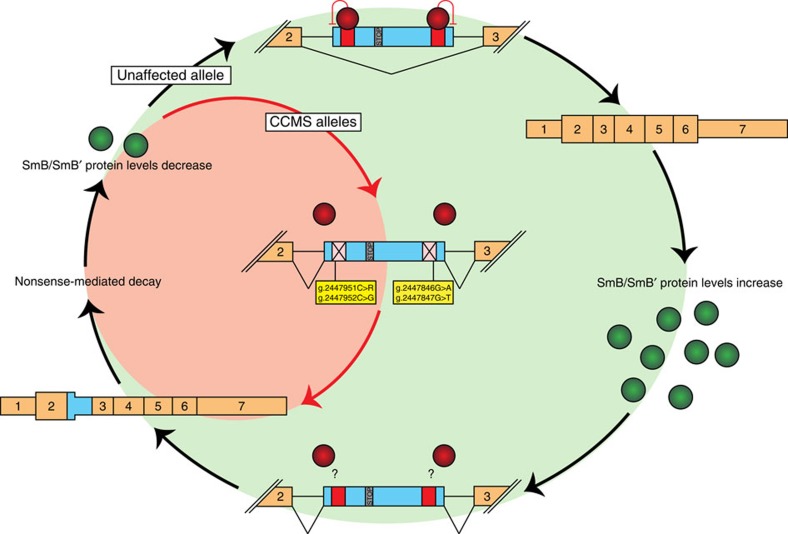
A model of disrupted *SNRPB* regulation in CCMS. Unknown repressor proteins (red circles) bind the ESS regulatory sequences (red squares) in the alternatively spliced exon (in blue) of *SNRPB*. Their binding leads to exclusion of this alternative exon, and thus an increase of SmB/SmB′ protein levels. Higher levels of these proteins then favour inclusion of the alternative exon, by an unknown mechanism, leading to NMD and a reduction of SmB/SmB′ protein levels. In alleles mutated in CCMS patients, the binding of repressor proteins is thought to be abolished or reduced due to the mutations present in the regulatory sequences. This leads to continued inclusion of the alternative exon, and reduced SmB/SmB′ protein levels due to NMD.

Collectively, these results implicate the deregulation of *SNRPB* expression as the main disease mechanism for CCMS. Mutations in the alternative PTC-containing exon cluster at two sites, which overlap with known exonic splicing silencers (ESSs)^22^. In an experiment by Saltzman *et al.*^18^, deletion of both of these regions resulted in increased inclusion of the alternative exon in HeLa cells. Our results support the functional significance of these ESSs, which are perfectly conserved across placental mammals (Fig. 3 and Supplementary Fig. 3), and suggest that the identified mutations weaken their silencing function. This would lead to the observed increase in the inclusion of this exon in CCMS, which is presumably the cause of the decreased overall *SNRPB* expression seen in patient cells (Fig. 4).

The mutations identified in the alternative exon appear to cause a reduction in the amount of SmB/B′ that is consistent with a hypomorphic, but not a null, allele. qRT–PCR experiments in three patients show a narrow range of total *SNRPB* expression (0.53–0.66 relative to controls). In the minigene experiment, exclusion of the alternative exon was not eliminated in mutant transcripts, but occurred 20–22% of the time. We also have evidence that null alleles might result in a more severe phenotype as one patient without an alternative exon mutation has a 5′ UTR mutation predicted to result in a null allele causing haploinsufficiency (Supplementary Discussion). This patient’s phenotype was more severe than the remainder of the cohort, with only five pairs of poorly ossified ribs, a poorly ossified spine, cystic hygroma and multiple pterygia (Supplementary Table 1). Since none of the other patients carry truncating mutations in the gene (which would be much more likely to occur by chance than point mutations at two specific loci), and truncating mutations in or deletions encompassing *SNRPB* have not been reported^23^,^24^, we suggest that *SNRPB* haploinsufficiency may cause a more severe and likely lethal phenotype that is distinct from classic CCMS.

CCMS joins a growing list of developmental disorders caused by mutations in core spliceosomal genes. Of particular interest are those with an overlapping craniofacial phenotype, such as Nager syndrome and the *EFTUD2*-related disorders^25^,^26^,^27^. Interestingly, all of the above are caused by dominant mutations that are predicted to reduce expression of a component of the major spliceosome. It is known that the abundance of the spliceosomal machinery influences AS^28^,^29^. In the case of *SNRPB*, RNAseq experiments have shown that specific AS exons are more sensitive to changes in SmB/B′ levels^18^. Among genes containing such exons, nucleic acid binding and RNA processing genes are over-represented. The *SNRPB* mutations presented here are therefore predicted to cross-regulate AS and expression of downstream genes. However, it is perplexing that a spliceosomal deficiency could cause such a strikingly specific phenotype. It may be that this deficiency affects a small number of transcripts that are particularly sensitive to spliceosomal protein levels. The identity of such transcripts remains speculative, however, animal models suggest that craniofacial abnormalities commonly found in CCMS are likely due to abnormal cell proliferation^30^, whereas the rib abnormalities have long been postulated to be a consequence of abnormal cartilage formation^31^. Given the common craniofacial phenotypes associated with the above-mentioned disorders, it is possible that a common gene or network of genes is perturbed in all of these. Another possible explanation for the specificity of the CCMS phenotype is that the spliceosomal deficiency is exacerbated in a critical tissue or developmental stage owing to increased demand for spliceosomal activity. Studies of the two spliceosome-associated disorders spinal muscular atrophy and retinitis pigmentosa have shown that the retina and spinal cord, tissues that appear to be sensitive to a spliceosomal deficiency, show increased demand for spliceosomal proteins^32^,^33^.

Our study highlights the importance of accurate AS in development, alludes to the broad network of splicing regulation, and demonstrates the regulatory and developmental importance of a highly conserved regulatory element. The alternative exon of *SNRPB* has high conservation at the nucleotide level throughout placental mammals (average GERP score 4.08), although to a lesser extent than the ultra-conserved elements (Supplementary Fig. 3). In general, shorter human conserved elements are conserved among mammals, but not with other species^3^. It has been suggested that evolution of these elements is ongoing in vertebrates, and that specific specializations may reflect clade-specific adaptive regulatory changes^3^. It is then possible that auto-regulation of *SNRPB* has evolved in mammals with the function of guiding specific cellular and developmental processes. Broadly, we therefore speculate that NCEs may have a significant role in regulating the phenotypic variation on which natural selection acts to drive the evolution of complex and highly integrated traits.

## Methods

### Patients

A cohort of 10 CCMS families was assembled through the Finding Of Rare disease GEnes (FORGE) Canada Consortium (now called Care4Rare). All patients provided informed consent, and the study was approved by and complies with the ethical regulations of the institutional review board at the University of Calgary. An experienced clinical geneticist was responsible for each diagnosis of CCMS. Exclusion criteria included absence of micrognathia and posterior rib gaps. Other variable features include scoliosis, short stature, conductive hearing loss and congenital heart defects. Although intellectual disability is reported to be a common feature of CCMS, this was not prevalent in our cohort (Supplementary Fig. 2 and Supplementary Table 1). Family A had a sibling recurrence with unaffected parents, families E and F had parent–child transmission, and the seven remaining cases were sporadic (Supplementary Fig. 1).

### Exome sequencing

DNA was extracted from whole blood. Exome sequencing was performed for six unrelated cases and seven family members at the McGill University and Génome Québec Innovation Centre. The SureSelect 50 Mb Human All Exon kit (Agilent) was used for exon capture; v3 was used for families A, B and C, and v5 was used for families D, E and F. Captured regions were sequenced on a HiSeq 2000 sequencer (Illumina) with 100 bp paired-end reads. Reads were aligned to the hg19/GRCh37 human reference sequence using the Burrows-Wheeler Aligner^34^, and indel realignment was done with GATK^35^. Duplicate reads were then marked using Picard ( http://picard.sourceforge.net/) and excluded from downstream analyses. Coverage of consensus coding sequence (CCDS) bases was assessed using the GATK, which showed that samples had on average >94% of CCDS bases covered by at least 10 reads, and >90% of CCDS bases covered by at least 20 reads. Single-nucleotide variants and short insertions and deletions were called with SAMtools mpileup^36^ with the extended base alignment quality adjustment (-E). Only variants that were supported by ≥20% of reads were returned. These were annotated using both Annovar^37^ and custom scripts to identify whether they affected protein-coding sequence, and whether they had previously been seen in the 1,000 genomes data set (April 2012), the National Institutes of Health Heart, Lung, Blood Institute, Grand Opportunity Exome Sequencing Project (NHLBI GO) exomes, or in ~700 exomes previously sequenced at our center.

To identify *de novo* variants in the probands of the three families for which trios were sequenced, we filtered out all proband variants seen in a parent or in the 1,000 genomes or NHLBI exome data sets, and manually reviewed remaining candidates. For family D a *de novo* 5′ UTR variant was seen that introduced a potential out-of-frame TIS in *SNRPB*. We used TIS miner ( http://dnafsminer.bic.nus.edu.sg/Tis.html) to predict the effect of this variant.

### Sanger sequencing

For all individuals in the cohort, Sanger sequencing of the alternative exon including the flanking intronic regions was performed. For patient D II-1, the 5′ UTR was sequenced to confirm the presence of the variant identified by exome sequencing. For patient G II-3, the coding regions, including the flanking intronic sequences, and the UTRs of *SNRPB* were sequenced. Primers were designed with Oligo 6 (Molecular Biology Insights). Sequences can be found in Supplementary Table 2. An amount of 2.5 μl of 50 ng μl^−1^ DNA was used in a 25-μl PCR using the HotStar Taq amplification system. Thermocycler conditions were as follows: 96 °C for 5:00, 35 cycles of 96 °C for 0:30, 58 °C for 0:30 and 72 °C for 0:30, and a final elongation step at 72 °C for 7:00. An amount of 5 μl was analysed on a 1% agarose gel. A quantity of 1.2 μl of 1/20 dilution of the PCR product was purified in a reaction with 1 μl ExoSAP-IT (Affymetrix) and 3 μl H_2_O The product of this reaction was added to a sequencing reaction with 2.2 μl H_2_O, 1.875 μl of 5 × sequencing buffer, 0.5 μl primer and 0.25 μl BigDye Terminator v1.1 (Life Technologies). Unincorporated nucleotides were removed from the sequencing reaction by passage through a Sephadex column. The products were then analysed on a 3130*x*L Genetic Analyzer (Applied Biosystems).

### Copy-number variant analysis by qPCR

qPCR of all exons of *SNRPB* was used to search for copy-number variants in patient G II-3. One microliter of 5 ng μl^−1^ DNA was used in a 20-μl reaction with 1 μl of 10 μM primer mix, 10 μl of SYBR Green (Life Technologies) and 8 μl of H_2_O. Primer sequences can be found in Supplementary Table 3. Reactions were run on a 7900HT Fast Real-Time PCR System (Applied Biosystems). Cycling conditions were as follows: 95 °C for 10 min, 40 cycles of 95 °C for 15 s and 60 °C for 1 min, and a dissociation step with 95 °C for 15 s, 60 °C for 15 s, and 95 °C for 15 s. Relative expression was calculated using the ΔΔ*C*_t_ method^36^, with *ALB* used as a reference gene.

### Cloning

GeneArt fragments with mutations were ordered from Invitrogen. Primer sequences can be found in Supplementary Table 4 and GeneArt fragment sequences can be found in Supplementary Table 5. These, along with the miniSmB plasmid (a gift of Dr Benjamin Blencowe), described in ref. 18, were digested with XhoI and NotI and gel purified. Ligation was done using the Quick Ligation kit (NEB), transformed and selected on ampicillin plates. Minipreps were prepared from selected clones and sequenced at the McGill University and Genome Quebec Innovation Centre.

### Transfections

HEK293 cells were plated in 24-well plates at 40–50% confluency. The following day, cells were transfected with 0.25 μg of DNA and 0.75 μl of Fugene 6 (Promega) in 2 ml of DMEM+10% FBS. Eighteen hours later, cells were rinsed with Dulbecco’s PBS and lysed with 1 ml of TRIzol (Invitrogen). RNA extraction was done using the manufacturer’s protocol and the resulting purified RNA was resuspended in 20 μl of diethylpyrocarbonate (DEPC)-treated H_2_O. Transfection was performed three times.

### RT–PCR and analysis

Half of the RNA (10 μl) was treated with 2 units of DNase I (NEB) by incubation at 25 °C for 15 min, then 1 μl of EDTA 25 mM was added, followed by incubation at 65 °C for 10 min. One-eighth of this reaction (2.5 μl) was used in a 10 μl reverse transcriptase reaction using SuperScript III (Invitrogen), using the manufacturer’s protocol, with random primers and a reaction temperature of 50 °C. RNase H treatment was performed by adding 1 unit of the enzyme and incubating at 37 °C for 20 min. A quantity of 0.75 μl of cDNA was used in a 30 μl PCR, of which 10 μl was analysed on a 2% agarose gel and 20 μl was analysed on a BioAnalyzer (Agilent). Primer sequences are available in the Supplementary Material.

### Patient fibroblast culture and RNA extraction

Skin biopsies were collected from patient II-1 from family C, patient II-2 from family E and patient II-1 from family F. Three anonymous control fibroblast lines were obtained from The Centre for Applied Genomics. Fibroblasts were cultured at 37 °C in Amniomax media (Invitrogen) (15% Amiomax supplement, 0.5% glutamine and 0.005% fungizome). At confluency, cells were treated with 3 ml of Hank’s balanced salt solution (Invitrogen), then 1 ml of trypsin-EDTA (Invitrogen) and centrifuged at 1,100 r.p.m. for 10 min. Cells were then either resuspended in fresh media for growth of the next passage or used for RNA extraction. Total RNA was extracted using the RNeasy Mini Kit (Qiagen) according to the manufacturer’s protocol, with a DNase I digestion performed at the wash step (10 μl DNase I in 70 μl buffer RDD (PreAnalytiX). RNA was extracted from three subsequent cell passages.

### qRT–PCR primer design and efficiency testing

Primers were designed using Primer3 ( http://bioinfo.ut.ee/primer3-0.4.0/primer3/) to overlap exon–exon junctions to prevent amplification of genomic DNA. Sequences can be found in Supplementary Table 6. For each primer pair, efficiency was determined by tracing a standard curve of the *C*_t_ values of four serial dilutions of cDNA. Primers with efficiency between 90 and 110% were selected. The SNRPBaltexon_qRTPCR_F and R primers amplify the transcript including the alternative PTC-containing exon; SNRPBtotal_qRTPCR_F and R primers amplify all transcripts.

### qRT–PCR analysis of *SNRPB* expression

Two microlitres of RNA were used in a 20 μl reverse transcriptase reaction using SuperScript III (Invitrogen), according to the manufacturer’s protocol, with oligo d(T) primers. The resulting cDNA was diluted by one-fifth and used in a qRT–PCR. All qRT–PCRs had a 20 μl volume, with 1 μl cDNA, 1 μl of 10 μM primer mix, 10 μl of SYBR Green (Life Technologies) and 8 μl of H_2_O. Reactions were run on a 7900HT Fast Real-Time PCR System (Applied Biosystems). Cycling conditions were as follows: 95 °C for 10 min, 40 cycles of 95 °C for 15 s and 60 °C for 1 min, and a dissociation step with 95 °C for 15 s, 60 °C for 15 s and 95 °C for 15 s. Relative expression was calculated using the ΔΔ*C*_t_ method^36^, with *EIF1B* used as a reference gene. The experiment was performed three times. Statistical significance of observed differences was calculated with a Student’s *t*-test.

## Author contributions

D.C.L. performed variant filtering, validation by Sanger sequencing, qRT–PCR in patient fibroblasts, and was the primary manuscript author. T.R. performed the minigene experiment, contributed figs 3 and 4, and edited the manuscript. J.S. performed bioinformatic analysis and edited the manuscript. E.J.B., E.H.Z., E.G.L., B.N.C., J.P.T., D.R.M., E.P.K., J.H.-F., L.F., R.S. and F.P.B. were responsible for the diagnosis of CCMS in this cohort, critical discussion regarding the CCMS phenotype and edited the manuscript. The Care4Rare Canada consortium performed whole-exome sequencing in patients. A.M.I., R.E.L., J.M., L.A.J.-M., J.S.P. and F.P.B. were involved in study design, data interpretation and editing of the manuscript. F.P.B. conceived the study and edited the manuscipt.

## Additional information

**Accession codes:** Sequence data for CCMS patients have been deposited in the PhenomeCentral repository ( https://phenomecentral.org) under the following accession codes: P0000462 60_11-03316 (for Family A II-3), P0000456 60_10-8546 (for Family B II-2), P0000459 60_11-00920 (for Family C II-2), P0000463 60_12-2740 (for Family D II-1), P0000435 60_12-4200 (for Family E II-2) and P0000454 60_12-5745 (for Family F II-1).

**How to cite this article:** Lynch, D. C. *et al.* Disrupted auto-regulation of the spliceosomal gene *SNRPB* causes cerebro–costo–mandibular syndrome. *Nat. Commun.* 5:4483 doi: 10.1038/ncomms5483 (2014).

## Supplementary Material

Supplementary Information Supplementary Figures 1-3, Supplementary Tables 1-6 and Supplementary Reference
