# Supplementary material for: Disrupted auto-regulation of the spliceosomal gene SNRPB causes cerebro–costo–mandibular syndrome
Source: Nat Commun. 2014 Jul 22;5:4483. doi: 10.1038/ncomms5483 (PMC4109005; doi:10.1038/ncomms5483)
Supplement: Supplementary Information — Supplementary Figures 1-3, Supplementary Tables 1-6 and Supplementary Reference [file ncomms5483-s1.pdf]

Supplementary Information

Supplementary Figures

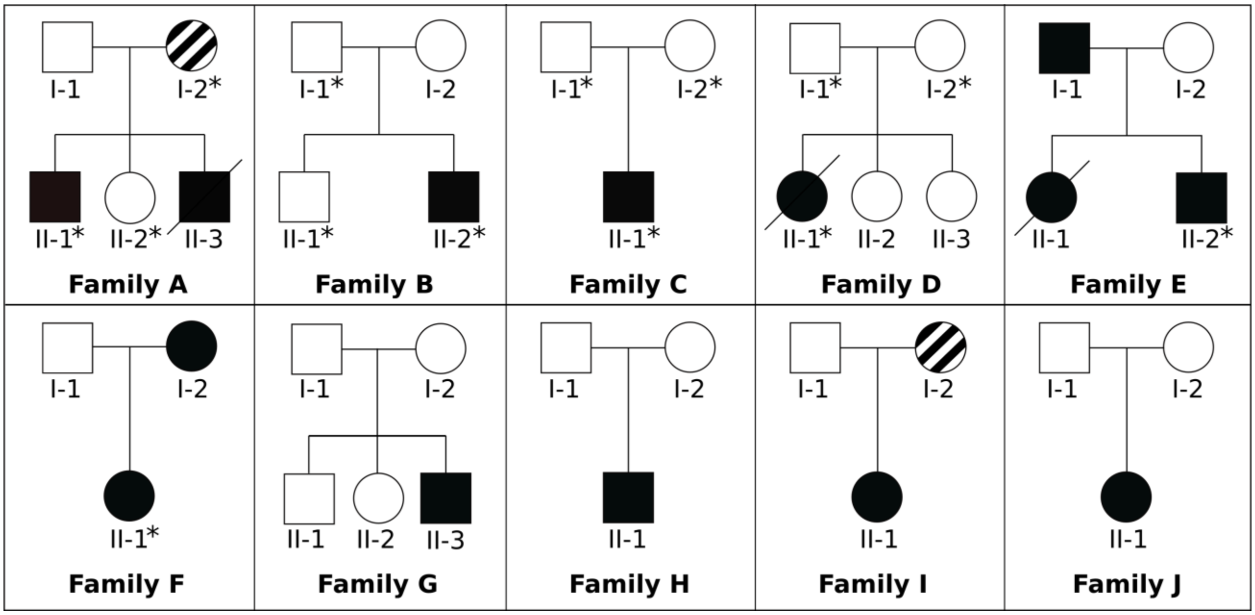

Supplementary Figure 1 Pedigrees of CCMS families A - J**.** Black symbols indicate affected individuals with at least micrognathia and posterior rib gaps. Striped symbols indicate *SNRPB* mutation carriers without CCMS but with a high arched palate. Asterisks denote individuals whose exomes were sequenced. All affected individuals except G II-3 have a *SNRPB* mutation

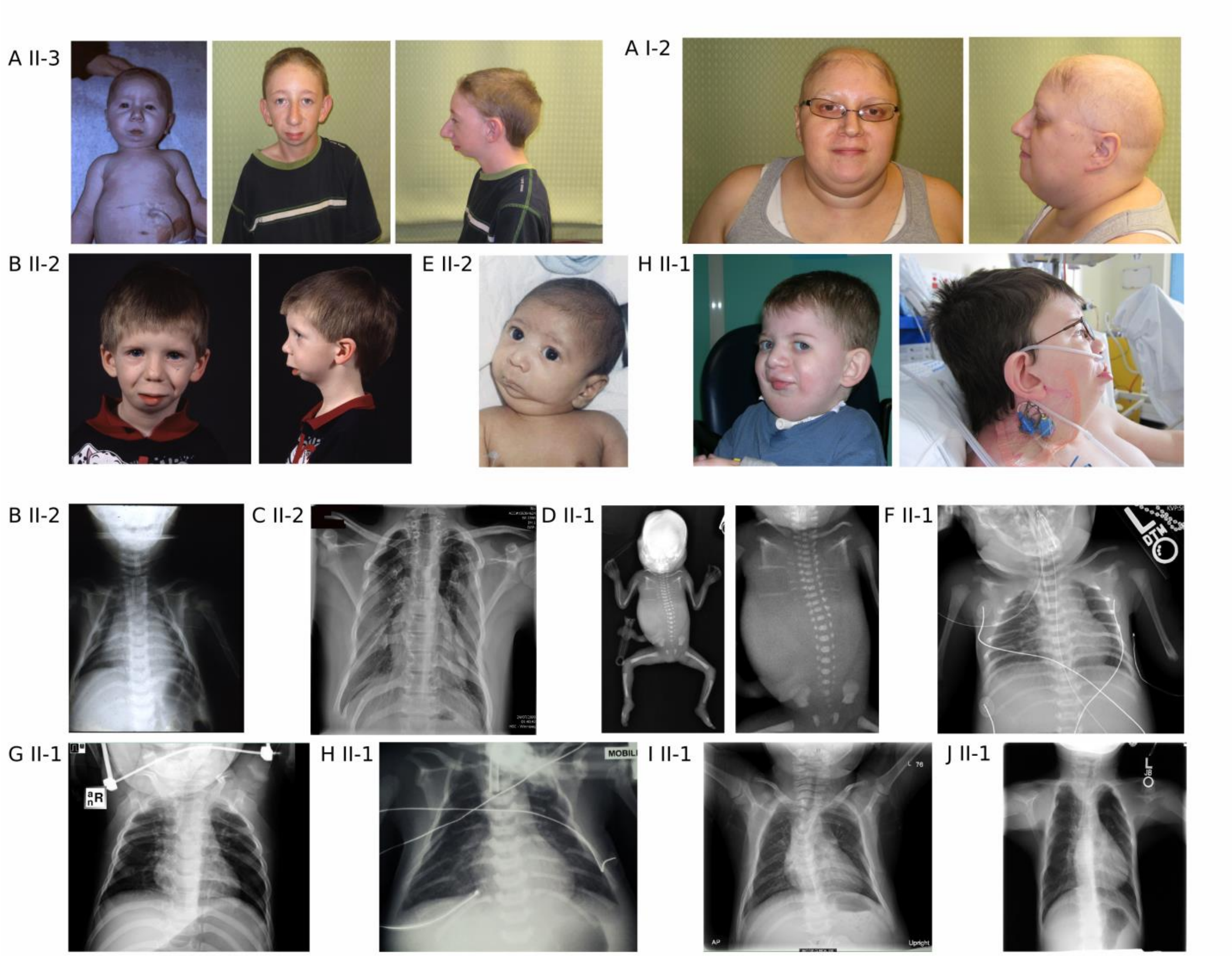

Supplementary Figure 2 Photographs and X-ray images of individuals with *SNRPB* mutations. A I-2 is not affected with CCMS, but shares a Chr20: 2447952C>G mutation with her affected son, A II-3. Her alopecia is due to chemotherapy. E II-2 had a cleft lip, which has been repaired. Micrognathia is seen in all individuals with CCMS. D II-1 had the most severe rib phenotype, with only five pairs of poorly ossified ribs. A narrow thorax and posterior rib gaps can be seen on all other X-ray image.

```
HUMAN_SNRPB          AGGGCTCTCA TGTTCTCCCC ACAGGTGTGT GAGGGGGGAG AGCTTGAGTT
SNRPB_CHIMP          AGGGCTCTCA TGTTCTCCCC ACAGGTGTGT GAGGGGGGAG AGCTTGAGTT
SNRPB_RHESUS         AGGGCTCTCA TGTTCTCCCC ACAGGTGTGT GAGGGGGGAG AGCTTGAGTT
SNRPB_MOUSE          AGGGCTCTCA TGTTCTCCCC ACAGGTGTGT GAGGGGGGAG AGCTTGATTT
SNRPB_RAT            AGGGCTCTCA TGTTCTCCCC ACAG..GTGT GAGGGGGGAG AGCTTGATTT
SNRPB_COW            AGGGCTCTCA TGTTCTCCCC ACAGGTGTGT AAGGGGGGAG AGCTTGATTT
SNRPB_DOG            AGGGCTCTCA TGTTCTCCCC ACAGGTGTGT AAGGGGGGAG AGCTTGATTT
SNRPB_ARMADILLO      AGGGCTCTCA TGTTCTCCTT ACAGGTGTGT AAGGGGGGAG AGCTTGATTT

HUMAN_SNRPB          TCTGGCCTCA TTGCCTATTT GTAAAGCTGT AGCCTAAAGG GCTCTCCCAA
SNRPB_CHIMP          TCTGGCCTCA TTGCCTATTT GTAAAGCTGT AGCCTAAAGG GCTCTCCCAA
SNRPB_RHESUS         TCTGGCCTCA TTGCCTATTT GTAAAGCTGT AGCCTAAAGG GCTCTCCCAA
SNRPB_MOUSE          TCTGGCCTCA TTACCTATTT GTAAAGCTGT AGCCTACAGG GCTCTCCTAA
SNRPB_RAT            TCTGGCCTCA TTACCTATTT GTAAAGCTGT AGCCTAAAGG GCTCTCCTAA
SNRPB_COW            TCTGGCCTCA TTGCCTATTT GTAAAGCTGT AGCCTAAAGG GCTCTCACAG
SNRPB_DOG            TCTGGCCTCA TTGCCTATTT GTAAAGCTGT AGCCTAAAGG GCTCTCCCAA
SNRPB_ARMADILLO      TCTGGCCTCA TTGCCTATTT GTAAAGCTGT AGCCTAAAGG GCTCTCCCAA

HUMAN_SNRPB          TGCAGCCTGG TCTGAAACTG CTTTAAGAAG CCTCTGACCC TCTTCAGGGT
SNRPB_CHIMP          TGCAGCCTGG TCTGAAACTG CTTTAAGAAG CCTCTGACCC TCTTCAGGGT
SNRPB_RHESUS         TGCAGCCTGG TCTGAAACTG CTTTAAGAAG CCTCTGACCC TCTTCAGGGT
SNRPB_MOUSE          TGCGGCCTGG TCTGAAACTG CTTTAAGAAG CCTCTGACCC TCTTCAGGGT
SNRPB_RAT            TGCAGCCTGG TCTGAAACTG CTTTAAGAAG CCTCTGACCC TCTTCAGGGT
SNRPB_COW            TGCAGCCTGG TCTGAAACTG CTTTAAGAAG CCTCTGACCC TCTTCAGGGT
SNRPB_DOG            TGCAGCCTGG TCTGAAACTG CTTTAAGAAG CCTCTGACCC TCTTCAGGGT
SNRPB_ARMADILLO      TGCAGACTGG TCTGAAACTG CTTTAAGAAG CCTCTGACCC TCTTCAGGGT

HUMAN_SNRPB          AAGTGCTCAG CTCCCCATGT GGGTTTGGGG CCAGTGATTT TTGTTGGCAT
SNRPB_CHIMP          AAGTGCTCAG CTCCCCATGT GGGTTTGGGG CCAATGATTT TTGTTGGCAT
SNRPB_RHESUS         AAGTGCTGAG CTCCCCATGT GGGTTTGGGG CCAGTGATTT TTGTTGGCAT
SNRPB_MOUSE          AAGTGCTCTG CTCCCCATGT GGGTTTGGGG CCAGTCATTT TTGTTGACAT
SNRPB_RAT            AAGTGCTCTG CTCCCCTTGT GGGTTTGGGG CCAGTCATTT TTGTTGGCAT
SNRPB_COW            AAGTGCTCAA CTCCCCTTGT GGGTTTGGGG CCAGTGATTT TTGTTGTCAT
SNRPB_DOG            AAGTGCTCAG CTTCCCGTGT GGGTTTGGGG CCAGTGATTT TTGTTGGCAT
SNRPB_ARMADILLO      AAGTGCTCAG CTCCCCATGT GGGTTTGGGG CCAGTGATTT TTGTTGACG.
```

Supplementary Figure 3 Alignment of the alternative PTC-containing exon in select placental mammals generated with ClustalW2[1]. The alternative exon in humans is highlighted in yellow. CCMS mutations are represented by blue triangles. The PTC is highlighted in red. Within the rectangles are ESS sequences identified by deletion mutagenesis of miniSmB[17]. The first ESS was also identified with FAS-ESS[21]. Note that the splice sites, PTC, and ESS sequences are conserved in all species represented

Supplementary Table 1 Supplementary data of the CCMS cohort

Family	Patient	Gender	Age	Thorax	Respiratory	Spine	Jaw	Palate	Neurological/ cognitive	Feeding	Height	Other	*SNRPB* mutation
A	I-2	F	NK	-	-	-	-	high arched	-	-	NK	breast cancer	g.2447952 C>G*
	II-1	M	dec. 3 mos.	bell-shaped MPRG	home O_2 deceased	scoliosis	PRS	high short	decreased tone	gastro-stomy	NK	down-slanting PFs	not tested
	II-3	M	13 y	bell-shaped MPRG	home O_2 in infancy	-	PRS	cleft	LD	gastro-stomy	NK	-	g.2447952 C>G†
B	II-2	M	17 y	bell-shaped MPRG 11 rib pairs	-	hypoplastic L5 pedicles	PRS	high arched	mild LD	gastro-stomy	50th %ile	down-slanting PFs dental caries	g.2447952 C>G‡
C	II-2	M	16 y	bell-shaped MPRG	restrictive lung disease	-	PRS	cleft	-	-	< 5th %ile	down-slanting PFs CHL	g.2447847 G>T‡
D	II-1	F	term. 20 wks	5 pairs of poorly ossified ribs 2 longer ribs on left side	NA	poorly ossified scoliosis	PRS	cleft	NK	NA	NA	ASD cystic hygroma choanal atresia nuchal webbing multiple pterygia	g.2451408 C>T‡
E	I-1	M	NK	abnormal	-	-	PRS	high short	-	-	3 – 10th %ile	-	g.2447952 C>G*
	II-1	F	dec. 3 mos.	NK	deceased	NK	PRS	NK	NK	NK	NK	NK	not tested
	II-2	M	19 y	MPRG	-	scoliosis	PRS	cleft	-	-	GH treatme nt 3rd %ile	CHL	g.2447952 C>G†
F	I-2	F	32 y	thin left 4th rib	-	scoliosis	PRS	cleft	mild delay	NK	NK	anal stenosis	g.2447951 C>G*

												CHL	
	II-1	F	8 mos.	Bell-shaped MPRG 10 rib pairs	thoracic insufficiency	-	PRS	cleft	-	NG tube	$< 5^{th}$ %ile	ASD echo-genic kidneys	g.2447951 C>G†
G	II-3	M	11 y	MPRG	asthma	-	PRS	cleft	-	NG tube	10 – 25th %ile	CHL absent teeth	-
H	II-1	M	12 y	MPRG 10 rib pairs	tracheostomy	scoliosis	PRS	cleft	-	-	$< 5^{th}$ %ile	cleft lip	g.2447952 C>G‡
I	I-2	F	32 y	normal	-	-	-	high arched	-	-	25th – 50th %ile	-	g.2447951 C>A*
	II-1	F	3 y	bell-shaped MPRG	tracheostomy until 23 mos.	-	PRS	cleft	-	gastro-stomy	30th %ile	ASD CHL strabismus hyperopia	g.2447951 C>A†
J	II-1	F	16 y	bell-shaped MPRG thin ribs missing left 12th rib	NK	-	PRS	cleft	-	-	56th %ile	CHL	g.2447846 G>A*

*inheritance unknown †familial mutation ‡*de novo* mutation

Abbreviations used: ASD = atrial septal defect CHL = conductive hearing loss F = female GH = growth hormone M = male LD = learning disability MPRG = multiple posterior rib gaps NA = not applicable NG = nasogastric NK = not known PF = palpebral fissure PRS = Pierre Robin sequence

Supplementary Table 2 *SNRPB* sequencing primers

SNRPB_ex1F	ACAGAGCAGCTCTCAGTACGGAT
SNRPB_ex1R	CCACTCCACAACAGACTCGG
SNRPB_ex2F	GACAAGAACGCCATGGGAAG
SNRPB_ex2R	CATCACCACCTCTATTGCTTGG
SNRPB_ex3F	AGGGCTATCTTGGGAAAGTTTG
SNRPB_ex3R	GGCAAAATCCAGAGGATGC
SNRPB_ex4F	GTCTTGGATGGAGGAAGAATG
SNRPB_ex4R	TCACAGGTTGGTCACTCTGGAC
SNRPB_ex5F	GTTATTTCCCACTGTCCTTCCC
SNRPB_ex5R	ACATTTGAGCCTAACACCCAGG
SNRPB_ex6F	CTGGGTGTTAGGCTCAAATGTC
SNRPB_ex6R	GCCCATGGGATATGATCTAAATG
SNRPB_ex7F	TCAGCCTCAGAGTAGTCCCTAGT
SNRPB_ex7R	TCATAGGCCACAAGGAGATAAA
SNRPB_ex7.2F	CCACGTTACCACAGACCTG
SNRPB_ex7.2R	TGTACAGATGCAAATGGGTAGT
SNRPB_altexon_F	GAGTCCAGCAAACCTTGCTCTC
SNRPB_altexon_R	GGGAGTTAGCCAATAACTTACGG
SNRPB_altexon3UTR_F	CTTGAGTTTCTGGCCTCATTG
SNRPB_altexon3UTR_R	TCTGTTGGCCCCTCTCTAATAC
SNRPB_altexon3UTR.2_F	CTGTGACCCAAGGTCAAGAAGT

SNRPB_altexonUTR.2_R	TTCATATCTGTTCCTCAAAAGCA
SNRPB_altexon3UTR.3_F	CCACTTTCCTATCCTGTTGCAG
SNRPB_altexon3UTR.3_R	AGCTTAAGGTCTTGCCAACATC

Supplementary Table 3 Quantitative PCR primers for determining *SNRPB* copy number

SNRPB_qPCR_ex1F	GAGGCTAGGCCTCTGAGGAG
SNRPB_qPCR_ex1R	GGTTCCCACTCCACAACAGA
SNRPB_qPCR_ex2F	TCATCCCTGTCCATTTCTCC
SNRPB_qPCR_ex2R	GAAGGTGCCAATGAAGATCC
SNRPB_qPCR_altexonF	AGTTGTGAAGGGCTGCTAGG
SNRPB_qPCR_altexonR	GGGAGAGCCCTTTAGGCTAC
SNRPB_qPCR_ex3bF	AAGGGAAGAGAAGCGAGTCC
SNRPB_qPCR_ex3bR	GGGAGGAGGTCCCTCTACTG
SNRPB_qPCR_ex4F	AGTTCCACTTGCTGGAGCTG
SNRPB_qPCR_ex4R	ACTCCTCACCTGTTGGGATG
SNRPB_qPCR_ex5F	CTGCTGCCACAGCCAGTAT
SNRPB_qPCR_ex5R	GGTCTCCTTATGGGCTCCTC
SNRPB_qPCR_ex6F	CCCACCTCCTGGTATGAGAC
SNRPB_qPCR_ex6R	TCACCACTGCAGGCACTTAC
SNRPB_qPCR_ex7F	CTCTTACTTCAGGCCTTCTTTGA
SNRPB_qPCR_ex7R	ATGAGTCTAGGGCCTTGGTG

Supplementary Table 4 Cloning primers for minigene experiment

ExonTrap_RTPCR_F	CTGCCCAGGCTTTTGTCAAACAG
ExonTrap_RTPCR_R	GTGCAGCACTGATCCACGATG
ExonTrap_seq_R	GTCGGTGCGGTGCTCTAT

Supplementary Table 5 GeneArt fragments

Chr20:2447847G>T	CGACTCGAGGAGGATGGGAATGGATTGAGTGGGTGG<br>GCCAAGCAATAGAGGTGGTGATGTAGCTAGTTGGTTC<br>TTCTCTGTTTATTCTCCAGTTTTAGAGGGCAGACTTAA<br>ATGGGGACATTGAACGCTAAGAAATCTTTTTGAGTCC<br>AGCAAACCTTGCTCTCTAGTCTAGCAAAGTACAAGTT<br>GTGAAGGGCTGCTAGGCTTTTTGTTTCTCTCACTTTGC<br>GTGCCCCTGGGTACTGGGGTAAAGGGCTCTCATGTTCT<br>CCCCACAGGTGTGTGAGGGGGGAGAGCTTGAGTTTCT<br>GGCCTCATTGCCTATTTGTAAAGCTGTAGCCTAAAGG<br>GCTCTCCCAATGCAGCCTGGTCTGAAACTGCTTTAAGA<br>AGCCTCTGACa*CTCTTCAGGGTAAGTGCTCAGCTCCC<br>CATGTGGGTTTGGGGCCAGTGATTTTTGTTGGCATTTA<br>TACAAACAATTGGAGATATTGTCTACTTTGCTACAATT<br>CCGTAAGTTATTGGCTAACTCCCTTGCCTGCGGCCGCT<br>TA
Chr20:2447951C>G	CGACTCGAGGAGGATGGGAATGGATTGAGTGGGTGG<br>GCCAAGCAATAGAGGTGGTGATGTAGCTAGTTGGTTC<br>TTCTCTGTTTATTCTCCAGTTTTAGAGGGCAGACTTAA<br>ATGGGGACATTGAACGCTAAGAAATCTTTTTGAGTCC<br>AGCAAACCTTGCTCTCTAGTCTAGCAAAGTACAAGTT<br>GTGAAGGGCTGCTAGGCTTTTTGTTTCTCTCACTTTGC

	GTGCCCCTGGGTACTGGGGTAAAGGGCTCTCATGTTCT<br>CCCCACAGGTGTGTGAGG**c**GGGAGAGCTTGAGTTTCT<br>GGCCTCATTGCCTATTTGTAAAGCTGTAGCCTAAAGG<br>GCTCTCCCAATGCAGCCTGGTCTGAAACTGCTTTAAGA<br>AGCCTCTGACCCTCTTCAGGGTAAGTGCTCAGCTCCCC<br>ATGTGGGTTTGGGGCCAGTGATTTTTGTTGGCATTTAT<br>ACAAACAATTGGAGATATTGTCTACTTTGCTACAATTC<br>CGTAAGTTATTGGCTAACTCCCTTGCCTGCGGCCGCTT<br>A

* point mutations are in bold and underlined

Supplementary Table 6 Quantitative RT-PCR primers

SNRPBaltexon_qRTPCR_F	TCAGAAAGATCAAGTGTGTG
SNRPBaltexon_qRTPCR_R	TAAAGCAGTTTCAGACCAGG
EIF1B02_qRTPCR_F	TACTGTTCAGGGCATTGCAG
EIF1B02_qRTPCR_R	CCTCTCCGTATTCAGGATGTTC
SNRPBtotal_qRTPCR_F	AAGCAGAAAGGGAAGAGAAG
SNRPBtotal_qRTPCR_R	CGAGCAATACCAGTATCTTTG

Supplementary Reference

1. Larkin, M. A *et al*. Clustal W and Clustal X version 2.0 *Bioninformatics* **23,** 2947-2948 (2007).
